# The Effects of the New Therapeutic Treatments for Diabetes Mellitus on the Male Reproductive Axis

**DOI:** 10.3389/fendo.2022.821113

**Published:** 2022-04-20

**Authors:** Carla Pelusi

**Affiliations:** ^1^Division of Endocrinology and Diabetes Prevention and Care, IRCCS Azienda Ospedaliero-Universitaria di Bologna, Bologna, Italy; ^2^Department of Medical and Surgical Sciences (DIMEC), Alma Mater Studiorum University of Bologna, Bologna, Italy

**Keywords:** diabetes mellitus, GLP 1 analogues, DPP4 inhibitors, SGLT2 inhibitors, male function

## Abstract

One of the complications of chronic hyperglycemia and insulin resistance due to type 2 diabetes mellitus (T2DM) on the hypothalamic-pituitary-gonadal axis in men, is the high prevalence of hypogonadotropic hypogonadism, which has been recently defined as functional hypogonadism, characterized by low testosterone associated with inappropriately normal gonadotropin levels. Although the pathophysiology of this hormonal imbalance may be related to several factors, including glycemic control, concomitant sleep apnea, insulin resistance, the main role is determined by the degree of central or visceral obesity and the consequent inflammatory state. Several drugs have been developed to treat T2DM such as glucagon-like peptide-1 receptor agonists, dipeptidyl peptidase 4 inhibitors, and sodium-glucose co-transporter 2 inhibitors. All appear to be effective in ameliorating blood glucose control, by lowering inflammation and body weight, and most seem to reduce the risk of micro- and macrovascular damage as a consequence of uncontrolled diabetes. A few studies have evaluated the impact of these drugs on gonadal function in T2DM patients with hypogonadism, with promising results. This review summarizes the main current knowledge of the effects of these new antidiabetic drugs on the hypothalamus–pituitary–gonadal axis, showing their potential future application in addition to glucose control in dysmetabolic male patients.

## Introduction

T2DM is a complex disease that is increasing worldwide. It is characterized by impaired glycemic metabolism leading to vascular and neuronal complications, in part due to the activation of several pathways inducing oxidative stress and mitochondrial damage (1). The male reproductive system is one of the targets of this disease, as shown by several studies on diabetic patients describing abnormalities of the hypothalamus–pituitary–gonadal axis (HPG), and particularly of the testicular activity and spermatogenesis, and erectile and ejaculatory function (2). The increasing prevalence of T2DM in young men seems to be partly responsible for the global decline in male fertility. Up to 40% of men with obesity and/or T2DM have co-existing hypogonadism (3), thus strengthening the important relationship between reproduction and metabolism. Although the mediators of this interaction are poorly understood, finding target therapies is essential in order to maintain adequate glycemic control for the management of co-existing metabolic complications such as hypogonadism (2). T2DM damage seems to be related to the duration of the disease, and to the type of glycemic control obtained with medication. Although there are several antidiabetic drugs aimed at appropriate glycemic levels, and thus effective in minimizing or preventing the appearance or development of T2DM-related complications (4), their effects on the male reproductive system are still under investigation.

This review focuses on the data available on the effects of the new antidiabetic drugs on the HPG axis. These include glucagon-like peptide receptor agonists (GLP1-RAs) and dipeptidyl peptidase 4 inhibitors (DPP4is), whose mechanisms of action, albeit different, are mainly related to the action of the glucagon-like peptide 1 (GLP-1). GLP1-RAs induce supra-physiological concentrations of ligands that stimulate the peripheral and central GLP-1 receptors (GLP-1R), whereas DDP-4is reduce the degradation of endogenously released GLP-1. Furthermore, few evidence of sodium-glucose co-transporter 2 inhibitors (SGLT2is), that through the inhibition of renal glucose reabsorption induce amelioration of glucotoxicity and inflammation.

## GLP1 General Function and Its Effect on HPG Axis

GLP-1 is an incretin, which is a hormone produced by the small intestine cells in the distal ileum and colon in response to food intake, whose levels are low in T2DM patients (5). This molecule exerts pleiotropic actions on the metabolism, as it increases glucose-dependent insulin secretion by pancreatic β-cells, decreases glucagon secretion, slows gastric emptying, and increases satiety (6). In animal models, it has also been shown to promote β cell mass expansion (7). GLP-1 binds and activates the GLP-1R, which is mainly expressed in pancreatic β-cells, but extra-pancreatic sites have been described as well, such as muscle cells, adipocyte, hypothalamic nuclei, pituitary and testicular gland, to cite some. GLP-1 has been shown to favor weight loss and to induce satiety by influencing brain regions at hypothalamic levels and other central appetite centers involved in the regulation of feeding. It has also a direct effect on gastric distension and peripheral vagal nerve activation causing satiety (8). Moreover, additional GLP-1 effects beyond energy homeostasis have been hypothesized. GLP-1 ameliorates the cardiovascular risk by directly or indirectly acting at multiple organ levels, reducing inflammation in the fat and other tissues, lowering blood pressure, increasing cardioprotection and favoring diuresis and natriuresis (9, 10).

Furthermore, GLP-1 seems to be implicated in the control of HPG function, as it modulates the activity of hypothalamic GnRH neurons and gonadal development (11–16). In *in-vitro* experiments on neuronal cell line, a concentration-dependent increase in luteinizing hormone- realizing hormone (LH-RH) release associated with intracellular cAMP accumulation was observed, after GLP-1 analog injections, supporting an activity of GLP-1 on GnRH neurons (11). In addition, it has been suggested that GLP-1 acts indirectly by stimulating *Kiss-1* gene expression and increasing kisspeptin release (13), known to be essential for fertility (14).

In animal studies, as well, GLP-1 analog administration induced GnRH neuron release *via* modulation of nitric-oxide and endocannabinoid pathways that regulate the GABAergic current in the postsynaptic GnRH neurons (12). Instead, reduced levels of GLP-1R mRNA in the pituitary have been shown (15), suggesting weak direct effects of GLP-1 at pituitary level. Acute GLP-1 treatment has been demonstrated to increase the amplitude of female rat preovulatory LH surge; however chronic exposure administration of a GLP-1R agonist was shown to reduce LH levels likely acting by stimulating or blocking the kisspeptin hypothalamic system level thus influencing GmRH and LH release (16). Moreover, in GLP-1R knockout (GLP-1R−/−) mice, a normal number and distribution of gonadotrophic cells in the anterior pituitary in both sexes were observed, without any anatomical abnormalities, thus supporting no direct action of GLP-1 at the pituitary level (16). Instead, male GLP-1R-/- mice, despite an impaired glucose metabolism, exhibited reduced testis and seminal vesicle weights, while females displayed a slight delay in the onset of puberty, despite normal steroid hormone levels and normal rate of reproduction (16).

In addition, studies in adult healthy men however have demonstrated that an infusion of GLP-1 after oral glucose ingestion during an euglycemic clamp reduces the number of pulsatile testosterone (T) secretion with a trend towards a longer T pulse duration by a mechanism independent of LH release, without altering the mean reproductive hormone levels (17). A similar study of nine healthy men who first had an oral glucose test, and then a 6 h continuous GLP-1R agonist infusion, resulted in a reduction in T levels at 30 minutes compared with baseline despite unaltered LH levels, and in mean T and LH levels. However, even if the authors reported a significant decrease in the number of T pulses and also a tendency for their increased duration, they found no impact of GLP-1 on the overall production of T (16). These data were confirmed by another study, which after intravenous infusion of high doses of GLP-1 in eighteen young healthy men, showed a reduction in food intake but no changes in serum levels of reproductive hormones, supporting no effect on LH and T pulsatility by GLP-1 administration (18).

Lastly, protein expression of GLP-1R was found in human and mouse testis, while increased mRNA content was demonstrated in human testis as well as in Leydig and Sertoli cell lines of mice (19, 20), supporting Leydig cells as a novel potential target for GLP-1.

Therefore, all these data may support the involvement of GLP-1 on the HPG axis and its potential impact on the male gonadal function.

## Glucagon-Like Peptide-1 Receptor Agonists: Evidence for Their Potential Effect on Male HPG Axis

Glucagon-like peptide-1 receptor agonists (GLP1-RAs) are currently used to treat obesity/T2DM. As previously reported (1–3), these disorders are frequently associated with an abnormal reproductive axis, at least in part due to the obesity induced suppression on the HPG axis, thereby interfering with gonadal function. Of the GLP1-RAs currently on the market, liraglutide has the most data on the male reproductive function. It has 97% homology to human GLP-1 and is characterized by a prolonged half-life to 13 h and is associated with progressive and sustained weight loss (21, 22). However, other GLP1-RAs include exenatide, semaglutide and dulaglutide, which differ in terms of molecular structure, doses and titrations, and pharmacokinetics (short and long acting agents) (23), glucose lowering efficacy, and mean body weight reduction (24). Several preclinical and human observational and interventional studies with different GLP1-RAs, have shown the potential direct impact of GLP-1 on the HPG axis, demonstrating improved T levels or sexual function (See **Tables 1** and **2**). In a retrospective study, Giagulli and colleagues (30) showed for the first time that the addition of liraglutide to the standard therapy of metformin and T, in obese men with T2DM and overt hypogonadism and with a poor response in terms of glycemic target, resulted in a significant improvement in the body weight and distribution of the glucose and lipid profile, and of the erectile function evaluated with the International Index of Erectile Function score. These results thus suggest the potential positive effects on metabolic and vascular function in these subjects. In a multicenter 12-week observational study conducted on middle- aged men with T2DM and obesity with low T, results showed that exenatide, at a dose of 10 μg twice daily, combined with metformin treatment had a better effect on serum T levels than glimepiride combined with metformin. The increase in T levels and amelioration of sexual function were closely related to the changes in body weight and waist circumference, thus supporting the potential positive effect of exenatide on the male reproductive system (34). In contrast to the previous studies, Graybill and colleagues showed no improvement in T in a prospective cohort study of 51 men starting exenatide extended release, at a dose of 2 mg once weekly, for T2DM for 6 months despite a reduction in weight and HbA1c (33). However, after analyzing the data and categorizing the population according to baseline T levels and the obtained improvement in HbA1c > 1%, a significant hormonal difference was observed, substantially supporting the potential benefit of these drugs on male reproductive function. In a prospective randomized open-label study on 30 obese middle-aged men with functional hypogonadism who had been poor respondents to lifestyle modifications, the effects of liraglutide and transdermal T replacement therapy for 16 weeks were compared. T significantly increased in both arms with improved sexual function. However, liraglutide was shown to be superior to T replacement therapy in improving T levels and overall metabolic abnormalities, thus suggesting that GLP1-RAs therapy has an overall health benefit for men with low T levels and metabolic dysfunction (31).

**Table 1:** Preclinical principal studies results on the effects of GLP1.

Drug	Study, ys	Subjects	Main findings
GLP1	MacLusky NJ, 2000 (16)	GLP-1R– /– mice	GLP-1 signaling does not result in a major reproductive behavioral deficit.
			In males, developmental masculinization apparently occurs normally, consistent with their normal growth curves and apparently normal reproductive performance in the breeding program.
			At autopsy, male GLP-1R–/– mice exhibited significant decreases in adrenal, testis and seminal vesicle weights compared with control animals,, suggesting GLP-1 role in the reproductive system
	Jeibmann A, 2005 (17)	Healthy men	GLP-1 i.v. infusion reduce the pulsatile component of T secretion by a mechanism independent of LH release
GLP1 RAs	Zhang E, 2015 (25)	HFD-induced obese mice	After 8-week exenatide treatment, sperm motility and activity were significantly increased when compared with the saline control in HFD group
DPP4is	Ayoub NN, 2015 (26)	STZ induced diabetic rat model	Even if sitagltin induced an amelioration of T levels, sitagliptiin showed markedly histopathologic changes in testis, epididymis and seminal vesicle.
SGLT2	Uthman L, 2019 (27)	Human cell	Emapglifozin and Dapaglifozin restore NO bioavailability by inhibiting ROS production
	Assaly R, 2018 (28)	T2DM rat model	Empaglifozin shows favorable effect on erectile function in diabetic rats mediated by an improvement of nitrergic relaxation of erectile tissue.

GLP-1R– /–, GLP 1 R knockout mice; T, testosterone; HFD, high-fat diet; STZ, streptozotocin; NO, nitric oxide; ROS, reactive oxygen species.

GLPI RAs, DPP4is and SGLT2 on male HPG male axis.

**Table 2:** Human studies on the effects of GLP1 RAs, DPP4is and SGLT2 on male HPG male axis.

	Author, yrs	Subjects	Study	Drug	Other medicine	Treatment period	HbA1c pre (%)	HbA1c post (%)	T pre (ng/ml)	T post (ng/ml)	SHBG pre (nmol/L)	SHBG post (nmol/L)	IIEF pre	IIEF post	Comments and other results
**GLP1 RAs**															
	Fontoura, 2014 (29)	Obese	Case report	LIRA	/	4 months	/	/							LIRA cause interrupted sperm production
	Giagulli, 2015 (30)	Obese T2DM	Ob	LIRA	MET and T	12 months	8.3±0.3	7.3±0.3	4.66±6.4	4.82±5.73	37.1± 2.8	59.1± 2.2	14.6 ± 1.7	19.9 ± 2.0	LIRA plus T and MET allows to achieve normal T levels as well as to reach glycaemic target and to lower weight, with ED improvement
	Jensterle, 2019 (31)	Obese	RCT	LIRA	LIRA vs T	16 weeks	5.9 ± 0.8	5.3 ± 0.4	2.19 ± 0.4*	2.94 ± 1.2*	26.3 ± 13.6	29.3 ± 14.1	/	/	LIRA was superior to T in improving an overall health benefit
	Giagulli, 2020 (32)	Obese T2DM	Ob	LIRA	MET	12 months	8.2 ± 0.2	6.9 ± 0.3	2,62 ± 1,1	3.28 ± 0.33	34.6 ± 2.3	39.0 ± 1.8	15.1 ± 1.1	19.1 ± 1.2	LIRA body weight reduction of more than 10% was associated with amelioration of HbA1c and a significant T and ED improvement
	Giagulli, 2020 (32)	Obese T2DM	Ob	DULA	MET	12 months	8.2 ± 0.3	7.0 ± 0.2	2.59 ± 1.2	3.10 ± 0.37	34.5 ± 2.2	39.4 ± 1.7	15.1 ± 1.2	18.7 ± 0.9	DULA body weight reduction of more than 10% was associated with amelioration of HbA1c and a significant T and ED improvement
	Graybill, 2021 (33)	Obese T2DM	Ob	EXE	/	6 months	8.1 ± 1.5	7.4 ± 1.4	3.34 ± 1.26	3.39 ± 1.33	36 ± 15	41 ± 22	/	/	EXE had a neutral effect on T levels
	Shao, 2018 (34)	Obese T2DM	Ob	EXE	MET	12 months	8.4± 0.9	6.6± 0.9	3.79 ± 1.01	5.01 ± 1.01	18.5 ± 1.7	28.90± 1.5	/	/	EXE and MET short-term combined treatment increased significantly T levels
**DPP4is**															
	Hibi, 2011 (35)	T2DM	Case-report	SITA		3 months	N,A,	6.1% reduced	N.A.	3.72					Unusual decrease effect on semen quality
**SGLT2**															
	Giagulli, 2020 (32)	Obese T2DM	Ob	DAPA	MET	12 months	8.0 ± 0.3	6.5 ± 0.3	2.65 ± 1.1	2.96 ± 0.27	36.5 ± 1.5	39.8 ± 1.4	15.8 ± 1.5	18.8 ± 1.4	DAPA body weight reduction of more than 10% was associated with amelioration of HbA1c and a significant T and ED improvement

Ob, observational; RCT, randomized control trial; LIRA, liraglutide; MET, metformina; T, testosterone; DULA, dulaglutide; EXE, exenatide; SITA, sitagliptin; DAPA, dapagliflozin; ED, erectile dysfunction; N.A., not available; *converted original data from nmol/L in ng/ml.

In a retrospective study of adult obese male patients with uncontrolled type 2 diabetes mellitus, who complained of mild to moderate erectile dysfunction, the authors investigated the contribution of body weight and glycemic control to the reversibility of hypogonadism to eugonadism under different glucose-lowering medications, including GLP1-RAs such as liraglutide and dulaglutide. They demonstrated that losing weight may have a greater impact on androgens compared to improving glycemic control, particularly in the groups with liraglutide and dulaglutide, and with dapaglifozin (32). In addition, a recent double-blind, placebo-controlled randomized trial of the effect of dulaglutide on cardiovascular outcomes showed that the long-term use of dulaglutide was associated with a reduce incidence of moderate or severe erectile dysfunction in men with type 2 diabetes (36).

Although in healthy subjects the acute effect of GLP-1 infusion has led to contradictory results (17, 18), the majority of interventional studies on dysmetabolic patients support the beneficial effects of GLP1-RAs treatment on the male reproductive system. This discrepancy could be explained by the different types of acute vs chronic GLP-1 infusions, and above all by the administration of the treatment in different dysmetabolic vs healthy subjects who have a different glucose metabolism. The relationship between low T and metabolism disorders is complex, bidirectional and not yet well known. It involves several mechanisms mainly based on insulin resistance, adipose release of inflammatory cytokines, and different hormone metabolisms (37).

Studies using a high-fat diet (HFD) induced obesity mice model characterized by decreased serum T levels, impaired sperm quality, and increased testis inflammation, showed that exenatide administration was able to reduce body weight and improve the quality of sperm without increasing serum T levels but lowering the expression of proinflammatory cytokines (25). By contrast, a case report described the adverse effects of the GLP1-RA liraglutide on spermatogenesis in a 35-year-old man experiencing primary and idiopathic infertility for one year. His first spermiogram at baseline showed normal parameters, whereas after four months of liraglutide application, semen analysis for intrauterine insemination showed no sperm motility. After five months of discontinuation of liraglutide the semen analysis returned to normal values for all the parameters (29). The explanation of this phenomenon and the different data obtained in animal and human studies are still unclear and need further evaluations.

Overall, these data support the use of GLP1-RAs in functional hypogonadism in the context of obesity or T2DM. Although it has been hypothesized that the GLP1-RAs effects on T levels were mainly mediated *via* weight loss, direct GLP.1 interactions with the HPG axis cannot be excluded. It is also known that weight loss may differ between different types of GLP1-RAs. The amount of weight loss is variable according to the type of GLP1-RAs administered, for example moderate with exenatide and dulaglutide, and robust with semaglutide and liraglutide, thus possibly explaining the diverse impacts on T levels (5, 38, 39). Therefore, future studies should address the differential impact of GLP1-RAs on reproductive health mediated *via* weight loss and improvement of metabolic status as opposed to the direct tissue-specific effects of GLP1-RAs that go beyond the weight-lowering potential. In fact, in addition to the neuroendocrine impact, GLP-1 seems to have anti-inflammatory and antifibrotic effects in different peripheral reproductive tissues, such as the testes. Whether GLP1-RAs therapy alone is sufficient to reverse the suppression of gonadotrophins found in functional hypogonadism in men with obesity and/or T2DM still needs to be clarified.

Finally, to strengthen the GLP1-RAs potential benefit effect on HPG axis, in studies conducted in obese PCOS women treated with GLP1-RAs (liraglutide and exenatide), amelioration of insulin resistance, reduction of hyperandrogenemia, improvement of ovulation rates, and restored menstrual cyclicity were observed (40–42).

## DPP4 Inhibitors (DPP4is): Evidence on Their Effects on Male HPG Axis

DPP4is are a relatively new class of oral diabetes drugs, also known as gliptins. They work by blocking the action of enzyme dipeptidyl peptidase 4 (DPP-4) in order to prevent the rapid degradation of endogenously released GLP-1, thus favoring glucose regulation. Sitagliptin, saxagliptin, linagliptin, vildagliptin and alogliptin are the current DPP4is on the market, which are usually prescribed for T2DM subjects who have poor glycemic control with lifestyle and metformin (43). To date, few studies have evaluated DPP4is impact on male reproductive function, showing controversial results (see **Tables 1** and **2**). In diabetic male rats induced by streptozotocin treated with several antidiabetic drugs in order to evaluate their impact on the reproductive system, the authors reported a lack of sitagliptin effects on the structure and weight of the testis, epididymis and seminal vesicle as well as on T levels (26). Although the testis of sitagliptin-treated rats presented the highest number of spermatocytes in different mitotic stages, a decreased expression of estrogen and androgen receptors was observed in the epididymis and in seminal vesicles. In contrast, based on the beneficial data of gliptins on cerebral ischemic stroke and cardiac ischemia reperfusion, several studies have evaluated DPP4is anti-inflammatory effects in animal model of testis injury/toxicity. The role and mechanism of action of gliptin have been investigated in a model of testicular ischemia/reperfusion injury by testicular torsion/detorsion. Protective effects have been highlighted, mostly due to anti-oxidative stress, and anti-apoptotic and anti-inflammatory actions, with increased T levels (44, 45). In a rat model of testicular damage induced by doxorubicin, Ahmed et al. showed that sitagliptin significantly increased T levels and the antioxidant capacity, thus reinforcing the antioxidant and anti-inflammatory properties of gliptins (46). Evidence in mice has shown that both DPP4is improved sexual function through their positive effects on the endothelium, probably due to the increase in nitric oxide (NO) levels, the release of the vascular endothelial growth factor which induces vasorelaxation, or by preventing atherogenesis through the induction of several possible substrates such as GLP-1, SDF-1α, substance P, and pituitary adenylate cyclase-activating polypeptide, which may enhance gonadotropin release, and therefore improves sex steroid levels (47).

Instead, until now, only one male case-report on DPP4is effect on HPG axis has been reported with uncertain meaning. Hibi et al. (35) described a 39-year-old diabetic man treated with sitagliptin (50mg/daily) with an amelioration of his hemoglobin A1c values to adequate levels, but with no semen at sperm analysis in addition to low free-T levels. The discontinuation of DPP4is led to a recovery of semen volume as well as sperm concentration and motility. The same procedure was performed several times with similar results suggesting a negative impact of DPP4is on the diabetic male’s fertility, but without a clear explanation.

Differently, studies conducted in women with PCOS treated with DPP4is have shown a decrease in inflammatory cytokine expression and oxidative stress, and activation of the anti-apoptotic pathway, thus improving ovarian cycles and ovulation (48).

In summary, although the studies on this topic are scarce, a direct or indirect beneficial impact of DPP4is, even if mild, on testis function cannot be ruled out. As opposed to GLP1-RAs, DPP4is have less impact on body weight, however their anti-inflammatory effects might preserve testis damage, which is often observed in obese diabetic patients. There is insufficient information to draw any conclusions, however DPP4is use in T2DM patients with additional functional hypogonadism is plausible, with caution on their use in male subjects of reproductive age. Studies are needed to provide clear information on the impact of gliptins on the male reproductive system.

## Sodium-Glucose Co-Transporter 2 Inhibitors: Evidence of Their Effects on Male HPG Axis

Sodium-glucose co-transporter 2 inhibitors (SGLT2is) are another new class of effective anti-diabetic drugs for treating T2DM (49). SGLT2is are responsible for major glucose reabsorption in renal proximal tubules, and therefore their inhibition leads to a reduction in blood glucose levels by increasing glucose urinary excretion. However, the beneficial effects of SGLT2 inhibition extend beyond blood glucose control. In fact, new studies have shown that the inhibition of renal glucose reabsorption reduces blood pressure, ameliorates glucotoxicity, favors weight loss, and induces hemodynamic effects that lead to improved cardiovascular and renal outcomes in T2DM (50, 51). Also based on their multiple nonglycemic effects, SGLT2is have therefore been suggested as the best glucose-lowering drug for managing patients with T2DM with heart failure (52). The SGLT2is currently on the market are canagliflozin, dapagliflozin, and empagliflozin, as single-ingredient products or in combination with metformin (53). Besides reducing glucose levels, empagliflozin has been shown to ameliorate endothelial dysfunction and atherogenesis and to improve cardiac remodeling in diabetic apolipoprotein E–deficient mice and in an experimental model of metabolic syndrome, the obese ZSF1 rat (54). In addition, mice with T2DM treated with dapagliflozin for eight weeks demonstrated significantly less arterial stiffness, improvements in endothelial and vascular smooth muscle dysfunctions, and reductions in circulating markers of inflammation compared with non-treated diabetic mice (55). In humans’ similar results were also obtained where the acute treatment of subjects with T2DM with dapagliflozin significantly improved systemic endothelial function and reduced both renal resistive index and aortic stiffness (56).

Studies exploring the possible direct effects of SGLT2is on male reproductive are scarce and in part related to their efficacy in endothelial dysfunction, such as in patients with erectile dysfunction (ED) (see **Tables 1** and **2**). Uthman et al. (27) reported that empagliflozin and dapagliflozin restored NO bioavailability by reducing ROS generation in tumor necrosis factor–α-stimulated human coronary artery endothelial cells and human umbilical vein endothelial cells. In a T2DM rat model with ED, treatment with empagliflozin for four weeks followed by acute sildenafil significantly improved erectile response in diabetic rats compared to placebo. This was associated with an improvement in cavernosal nitrergic relaxation, suggesting a positive effect of empagliflozin on the nerve injury (28). Therefore, these findings suggest that gliptins have favorable effects on erectile function, although further investigations are needed to understand whether these effects are due to better glycemic control or to a reduction in the diabetes-associated inflammatory state, and/or alternatively to a direct effect on penile endothelial cells.

Furthermore, only one retrospective study has demonstrated an improvement in body weight with a parallel increment in T levels in 16 obese patients with uncontrolled type 2 diabetes mellitus and hypogonadism treated with dapagliflozin (32). The increase in T was explained as a consequence of the amount of weight lost and the reduction in inflammation (32).

Although data on the effects of SGLT2is on HPG axis and function are poor and there are no data on testis morphology, due to their multiple metabolic effects beyond glucose control, we might presume potential SGLT2is benefit, at least indirectly through the reduction of glucotoxicity and inflammation, on male reproduction in diabetic patients with functional hypogonadism.

Finally, the combined SGLT1/SGLT2 inhibitors are currently under investigation. They reduce glucose absorption in the gastrointestinal tract due to SGLT1 inhibition and reduce renal glucose reabsorption *via* the inhibition of both transporters, thus inducing increased GLP-1 levels (57). To date there are no data on the impact of this type of drug on the reproductive male system, however studies in PCOS women with licogliflozin, a dual SGLT1/2 inhibitor, showed an amelioration of hyperinsulinmeia and hyperandrogenism, but not of T levels, without any effect on body weight (58).

## Conclusions

**Figure 1:**
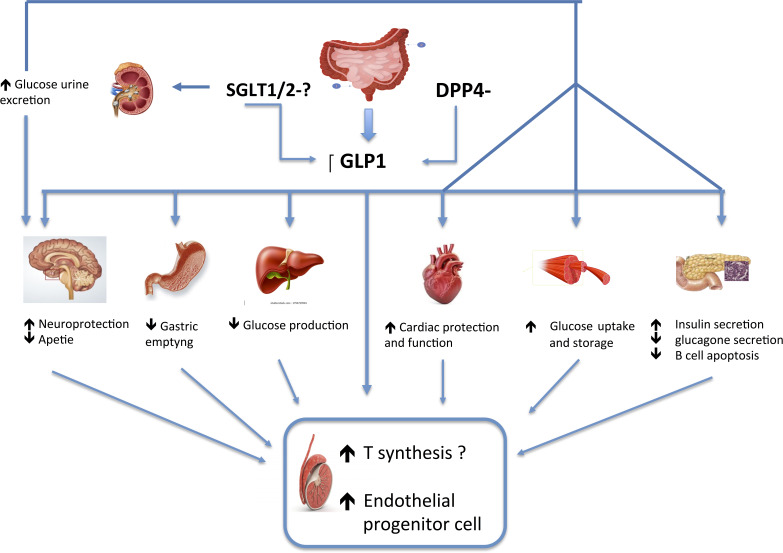
Effects of the new anti-diabetic drugs on male HPG axis.

There are few studies on the impact of these new classes of anti-diabetic drugs on the HPG axis and in particular on testicular function. However due to their mechanisms of action, it is plausible that they all could have direct or indirect beneficial effects on the male reproductive axis, mainly through the action of GLP -1 (**Figure 1**). Besides working on glucose control, the GLP1-RAs appear to be the most effective on losing weight, reducing inflammation, and on modulating testicular function, thus supporting their favorable application in male dysmetabolic patients with hypogonadism. Regarding DPP4is and SGLT2is, there are still unclear and scarce data available on their potential effect on HPG axis to draw any conclusion; however, it is plausible to hypothesize that these drugs would at least indirectly affect this condition by reducing glucotoxicity and inflammation. Furthermore, it is possible that this effect could be achieved by SGLT2is through weight loss.

Although there are still unclear data on how much improvement in hyperglycemia in diabetic patients is needed to affect T and the entire HPG axis, the identification of effective and early treatments may prevent irreversible organ-damage. All these antidiabetic treatment options have been shown to be capable of reaching a good glycemic target, so that therapy nowadays can be individualized and chosen according to patient’s risk factors, HbA1C levels, formulation and ease of use, costs, and potential side effects.

Therefore, future studies may benefit from an evaluation of these antidiabetic drugs and their comparisons in dysmetabolic patients with hypogonadism to verify their efficacy and potential different impact on the HPG axis. These information might help the physician in the selection of a more suitable drug based on the needs/complications beyond glucose control.

## Author Contributions

The author confirms full responsibility for the following: study conception and design, data collection,and interpretation of results, and manuscript preparation.

## Conflict of Interest

The author declares that the research was conducted in the absence of any commercial or financial relationships that could be construed as a potential conflict of interest.

## Publisher’s Note

All claims expressed in this article are solely those of the authors and do not necessarily represent those of their affiliated organizations, or those of the publisher, the editors and the reviewers. Any product that may be evaluated in this article, or claim that may be made by its manufacturer, is not guaranteed or endorsed by the publisher.
